# Necrotizing Fasciitis in Switzerland: Risk Factors and Clinical Outcomes Based on a 12-Year Retrospective Study

**DOI:** 10.3390/jcm15103689

**Published:** 2026-05-11

**Authors:** Annina M. C. Fürer, Athanasios Papanikolaou, Mihai Constantinescu, Parham Sendi

**Affiliations:** 1 Faculty of Medicine, University of Bern, 3008 Bern, Switzerland; annina.fuerer@bluewin.ch; 2 Department of Plastic, Reconstructive and Aesthetic Surgery, Inselspital University Hospital, 3010 Bern, Switzerland; 3 Department for BioMedical Research, Faculty of Medicine, University of Bern, 3008 Bern, Switzerland; 4 Institute for Infectious Diseases, University of Bern, 3001 Bern, Switzerland

**Keywords:** necrotizing fasciitis, soft tissue infection, polymicrobial infections, *Streptococcus* infections

## Abstract

**Background:** Necrotizing fasciitis (NF) is a rapidly progressing and life-threatening soft tissue infection. It commonly affects the extremities and is triggered by trauma, surgical wounds or compromised skin integrity. Although there have been advancements in diagnosis and treatment, NF continues to be a complex condition due to comorbidities and the wide range of involved pathogens. The aim of this study was to assess the epidemiology, risk factors, and outcomes of NF in Switzerland, focusing on the distinction between polymicrobial and monomicrobial infections and their associated management. **Methods:** This monocentric retrospective study analyzed 75 patients treated for intraoperatively confirmed NF from 2010 to 2022. Data collection included demographic and clinical variables, comorbidities, and laboratory results. Univariate and multivariate statistical tests were conducted to assess associations between mortality and different infection types. **Results:** NF was most commonly localized in the lower extremities (52% of cases). Nearly all patients (99%) had at least one comorbidity, with coronary heart disease, kidney disease, and liver disease being most frequently observed. Advanced age was associated with higher in-hospital mortality (OR (odds ratio) 3.80, *p* = 0.033, q = 0.500, 16 of 44 patients over 60 died), and the overall in-hospital mortality in the cohort was 27%. Kidney disease and smoking were associated with polymicrobial infections, with an OR of 6.60 (*p* = 0.016, q = 0.130) and 5.60 (*p* = 0.009, q = 0.130), respectively. Trauma was associated with monomicrobial streptococcal infections (OR 3.40, *p* = 0.029, q = 0.500), and showed a significant association with *Streptococcus pyogenes* infections (OR 8.10, *p* < 0.001, q = 0.004). **Conclusions:** This study highlights advanced age, trauma, kidney disease and smoking as factors associated with worse outcomes and specific infection patterns in patients with NF. These findings, derived from a Swiss patient population, underscore the importance of early identification of high-risk individuals to improve clinical outcomes. Recognizing these risk profiles may support clinicians in prioritizing rapid escalation of care for the patients most likely to benefit from early and aggressive intervention.

## 1. Introduction

Necrotizing fasciitis (NF) is a critical, soft-tissue infection characterized by rapid progression and significant mortality [1,2]. The extremities are the most common sites of infection [3].

NF typically arises following trauma or disruption of skin integrity [4]. The condition presents a clinical challenge due to its rapid progression and high mortality rate [5,6,7,8]. Known comorbidities associated with this disease include chronic kidney insufficiency, cardiovascular disease, obesity, diabetes mellitus, immunosuppression, liver cirrhosis, pulmonary diseases, malignancy, and illicit intravenous drug use [2,9,10,11,12]. Advanced age increases the risk of developing NF as well as the risk of death [6]. Additional factors associated with higher mortality include female sex, the extent of infection, delayed initial debridement, elevated creatinine and lactate levels, and organ dysfunction at the time of admission [10].

According to Morgan [13], NF can be classified according to its range of pathogen species, manifesting primarily in four types:

Type I NF, the most prevalent (55–80% [13,14]), is a polymicrobial infection (caused by two or more microorganisms) often resulting from a mix of anaerobic, aerobic, and facultatively anaerobic bacteria. This type predominantly affects individuals with compromised immune systems due to various comorbidities such as chronic renal failure, diabetes mellitus, and obesity, and typically occurs on the trunk and perineum. Reported mortality exceeds 32% [13,15].

Type II NF, caused mainly by Group A beta-hemolytic *Streptococcus* (GAS), represents a smaller percentage of cases (20–30% [13]) with a mortality rate of up to 67% [14] and is known for its aggressive nature and rapid progression. A notable rise in GAS NF cases has been observed since the 1990s, with mortality rates ranging significantly [13].

The rare form of type III NF (prevalence < 1% [14]) is primarily found in Asia, involves Gram-negative, often marine-related organisms like *Vibrio* species, with a particular threat posed by *Vibrio vulnificus*, especially in individuals with liver disease [13]. This type is associated with high mortality rates, with reported values reaching up to 43% [13,14,16].

Caused by fungal infections, type IV NF is the rarest form with a prevalence < 1% [14], predominantly affects immunocompromised patients [13,17], and has been reported to carry a mortality rate of 69% [18]. Even though the above-mentioned classification is well known and commonly used, other NF classifications have been proposed as well, including simplified systems that divide NF only into type I and type II. Additional approaches classify NF based on the anatomical site of infection, such as Fournier’s gangrene, which affects the perineal and genital region, or Meleney’s gangrene, which typically involves the postoperative abdominal wall [2,15,19].

Despite advancements in diagnostic techniques and management protocols, the morbidity and mortality associated with NF remain consistently high. This has led to the development of the Laboratory Risk Indicator for NF (LRINEC) score, aimed at facilitating early diagnosis and distinguishing NF from other less severe soft tissue infections [20]. However, its low sensitivity (59% at a cut-off of ≥6 points) has raised concerns about reliability [16,19,21] and is therefore better used as an adjunct rather than a definitive diagnostic instrument [21].

NF affects both sexes, although some studies suggest a male predominance with ratios up to 3:1 [2,6,14]. Mortality rates vary widely and can reach up to 76%, particularly in patients with comorbidities such as diabetes, chronic renal failure, or liver cirrhosis [4,6,10].

The global incidence of NF ranges from 0.3 to 15 cases per 100,000 population annually; however, this number is likely underestimated due to diagnostic challenges and underreporting. In the United States of America (USA), approximately 500–1000 cases are reported each year, corresponding to an incidence of 0.4 per 100,000 [22,23,24]. European countries report about 1 case per 100,000 annually [6,7,24]. In contrast, higher rates have been observed in Thailand (15.5/100,000) [22] and New Zealand (1.7/100,000 in 2006) [25,26]. Incidence data from African countries remain unavailable [27,28,29].

Similarly, geographical differences have been reported for mortality rates: 29% in the Netherlands [12], 21% in Taiwan [30], and in Nigeria, 9% in children and 17% in adults [31].

Despite apparent differences, such mortality rates, regardless of country-specific healthcare system characteristics, highlight the importance of early and accurate diagnosis as well as timely, effective treatment [22,23,32].

Regarding Switzerland, limited mortality data exist. One retrospective study (1999–2004, 13 cases) reported 15%, while a 2018 single-center study (2011–2017, 61 cases) found 28% [33,34]. This study expands these findings through a broader analysis of NF-related mortality.

Given the variations in the frequency of NF across different countries, attributed to differences in healthcare systems, reporting practices, and population health, it becomes evident that the incidence, management, and outcomes of NF can be influenced by regional factors. Switzerland, with its high-quality interconnected healthcare system, widespread access to medical facilities and efficient emergency services, presents a unique setting for the study of NF. The country’s short distances between medical institutions and prompt referral systems may contribute to differing incidence rates and outcomes for NF compared to regions with less accessible healthcare systems. This underscores the importance of regional investigations into NF, in order to understand how such factors may influence the disease’s impact.

This retrospective, monocentric study aims to evaluate NF within a university hospital setting in Switzerland. It focuses on the etiology, clinical characteristics, and risk factors associated with polymicrobial and monomicrobial infections. We investigate risk factors associated with all-cause mortality using univariate and multivariate analyses and assess potential patterns and correlations across different NF pathogens. Furthermore, the study evaluates the effectiveness of diagnostic and therapeutic strategies to improve patient outcomes and reduce mortality.

We hypothesize that mortality rates in Switzerland are lower than those reported in the literature and that a rate below 20% reflects a high standard of care provided by the Swiss healthcare system.

## 2. Materials and Methods

### 2.1. Study Design and Patient Population

This retrospective, monocentric study analyzed patients treated for NF at the Department of Plastic Surgery at the University Hospital Bern, Switzerland, from January 2010 to December 2022. Two principal investigators (A.F. and A.P.) conducted database searches using multiple patient databases that were formerly in clinical use at the University Hospital Bern, Switzerland. These include the clinical documentation systems IPDOS (Inselspital, University Hospital, Bern, Switzerland) and RAP (Inselspital, University Hospital, Bern, Switzerland).

Patients were identified through a comprehensive review of medical records, specifically targeting those with a clinical diagnosis or emergency referrals with severe soft tissue infection or reported suspicion of NF. This approach to data investigation was selected since NF cases have a clinical presentation of a severe soft tissue infection; however, not all severe soft tissue infections are NF. In order not to miss a case of NF, we began patient assessment from a more general diagnostic approach. Inclusion criteria required an intraoperatively confirmed diagnosis of NF, validated by intraoperative pathognomonic findings of necrotic tissue along the plane of the subcutaneous fat and superficial fascia, dishwater fluid and a loosened tissue plane with a positive finger probe. A total of 214 patients with severe soft tissue infections were initially assessed during the study period, based on corroborating clinical suspicion, reason for referral and clinical findings compatible with NF. The diagnostic strategy was the same for all patients, namely, intraoperative confirmation or exclusion of NF, and the management protocol was uniformly applied for all cases, namely, aggressive debridement to healthy tissues in the shortest possible timeframe.

### 2.2. Data Collection

Among the initially suspected cases of necrotizing fasciitis, the diagnosis could not be confirmed intraoperatively in 139 patients, and these cases were therefore excluded. These cases represented other severe soft tissue infections such as cellulitis or phlegmon, which can present with clinical features similar to necrotizing fasciitis. The exact diagnoses of these excluded cases were beyond the scope of this study.

**Chart 1:**
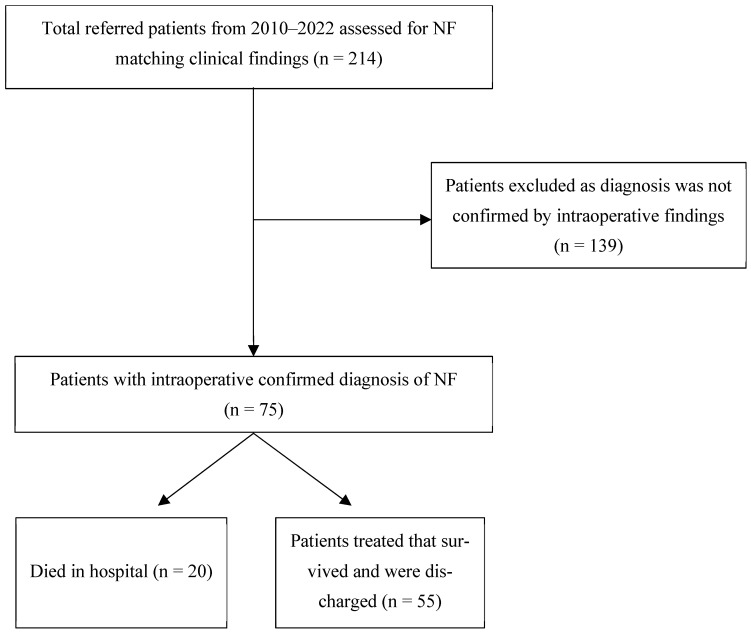
Patient population.

Seventy-five patients who underwent surgery were confirmed to have NF and were included in the final analysis (Chart 1). Retrospectively collected data from medical records included demographic characteristics, comorbidities, clinical findings, postoperative outcomes, and laboratory results.

### 2.3. Data Cleaning

Given that retrospective chart reviews may contain gaps in documentation, all records with less than 80% completeness were excluded to maintain data integrity and quality. Thus, if more than 20% of the data for a specific variable were missing (e.g., C-reactive protein values, leukocyte counts, and clinical parameters such as improvement of local inflammation or cessation of spreading), that variable was not analyzed further and was excluded from the analysis.

### 2.4. Patient Characteristics

Demographic data and comorbid conditions were assessed for each patient. The prevalence of comorbidities such as diabetes mellitus, coronary heart disease, liver disease, kidney disease, history of smoking, alcohol consumption, and drug abuse was noted.

### 2.5. Clinical Findings and Treatment Outcomes

**Figure 1:**
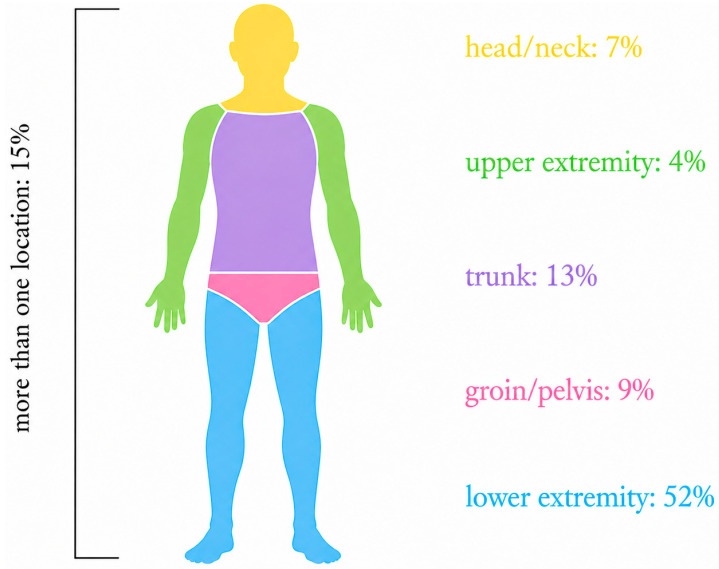
Localization of NF.

Data regarding clinical findings upon presentation and during hospitalization were collected, including the location of infection (Figure 1), pain level, and presence of specific symptoms known to occur in NF, such as pain out of proportion and “map-like redness” of the skin (Table 1). Surgical interventions, the number of surgeries per patient (Table 2), time from presentation to surgery (Table 2), antibiotic regimens, and changes therein (Table 3) were documented to analyze treatment patterns and efficacy. A single outlier case contributed to the median time of 3 h from presentation in the emergency room to surgery, involving a patient who was admitted for a different diagnosis and only later diagnosed and treated for NF while already hospitalized.

The postoperative outcomes were analyzed through several parameters, including wound closure during the hospital stay, recurrence of infection (defined as clinical deterioration and infection progress after the time point at which the infection was considered treated), in-hospital death (Table 4), and necessity for extremity amputation. These outcomes were assessed to evaluate the effectiveness of surgical and medical treatments provided to the patients.

### 2.6. Laboratory Findings

Documentation of laboratory findings focused on markers of infection and inflammation, primarily C-reactive protein (CRP) levels and leukocyte counts. The data collected included CRP levels upon admission, postoperatively, and at the time of recurrence or wound closure, as well as significant changes in leukocyte counts. The antimicrobial susceptibility testing was interpreted according to EUCAST guidelines.

### 2.7. Statistical Analysis

All statistical analyses were performed using the statistical software IBM SPSS Statistics (version 29.0, IBM Corp., Armonk, NY, USA). To analyze and summarize the qualitative data, descriptive statistics were calculated, including means, standard deviations (SD), and medians, while categorical variables were summarized with frequencies and percentages. A significance level of α = 0.05 was applied for all hypothesis tests.

We performed two stages of analysis for each of the questions 1–5:

Question 1: Which risk factors are associated with hospital death of the patient?

Question 2: Which risk factors are associated with polymicrobial infections?

Question 3: Which risk factors are associated with monomicrobial infections of type Streptococcus?

Question 4: Which risk factors are associated with infections of Group A, C, G or anginosus Streptococcus, respectively (as compared to infections with any other type of pathogen)? 

Question 5: More generally, which risk factors are associated with which pathogens?

Univariate analysis: For each research question, univariate analyses were performed to assess the association between individual risk factors and the respective binary outcomes. Given the limited sample size and the number of candidate predictors, the two continuous variables, age and BMI, were dichotomized for the univariate analyses as >60 years and >30 kg/m^2^, respectively. All other risk factors and dependent variables were binary. Therefore, the two-tailed Fisher’s exact test was used for the univariate analyses. To account for multiple testing, *p*-values were adjusted using the Benjamini–Hochberg procedure to control the false discovery rate (FDR) at 0.05 for each research question.

Multivariate analysis: Exploratory multivariable analyses were additionally performed for outcomes with sufficient numbers of events. Because conventional multivariable logistic regression was considered unstable in relation to the small sample size and the number of predictors, lasso-regularized logistic regression was used instead. In these exploratory models, age and BMI were retained as continuous variables, whereas all other predictors remained binary. For each dependent variable, models were fitted across a range of penalty values (λ), and the optimal λ was selected using 10-fold cross-validation. These multivariable analyses were considered exploratory and were not used to infer statistical significance or independent risk factor effects.

Artificial intelligence (AI) tools (ChatGPT based on GPT-5.2, OpenAI, San Francisco, CA, USA) were used for language editing, including grammar correction, typographical error correction, rephrasing, punctuation, reference list checking and the creation of Figure 1. All AI-assisted content was independently verified by the authors.

## 3. Results

In total, 39 men and 36 women were included in the analysis, with an age range of 13–92 (see Table 5). In our study cohort, 99% of patients (74 patients) had at least one known comorbidity (see Table 6). The three most common comorbidities were coronary heart disease, present in 61% of patients (46 patients), a history of kidney disease in 56% (42 patients) and liver disease observed in 43% (32 patients). Other comorbidities, such as diabetes mellitus, trauma history and lung disease, are listed in Table 6 for further reference. Furthermore, Table 7, Table 8 and Table 9 illustrate the pathogens, initial antibiotic therapy at clinical suspicion of necrotizing fasciitis, and the most common initial antibiotic combinations.

Anatomic location (see Figure 1): The lower extremity (blue) was the most common site of infection, occurring in 52% of cases (39 patients). The trunk (purple) was affected in 13% of patients (10 patients), followed by the groin/pelvis (pink) in 9% (7 patients), and the head/neck (yellow) region in 7% (5 patients). Involvement of the upper extremity (green) was rare, observed in only 4% of cases (3 patients). In addition, 15% of cases (11 patients) presented with infections at more than one anatomical location. In 11 patients with affected extremities, an amputation was required, and 2 of these patients died during their hospital course.

Risk factor analyses were performed according to the predefined research questions (Q1–Q5).

Q1: Hospital death

We identified an overall in-hospital mortality of 27% (20 in-hospital deaths, Table 4). In univariate analysis, patient age was associated with mortality during the hospital stay. The median age was 63 years (see Table 5), and for the sake of our analysis, we considered patients above 60 years of age as advanced age. Specifically, patients over the age of 60 displayed a markedly higher incidence of in-hospital death in comparison to younger patients, with 16 out of 44 patients over 60 dying during hospitalization (OR for hospital death was 3.80; 95% CI: 1.04–17.60, *p* = 0.033, q = 0.5), which was not statistically significant after adjustment for multiple testing.

Q2: Polymicrobial infections

In univariate analysis, patients with an established diagnosis of kidney disease exhibited a higher incidence of polymicrobial infections, with an odds ratio (OR) of 6.60 (95% CI: 1.30–65.80, *p* = 0.016, q = 0.130), and smoking was associated with polymicrobial infections, with an OR of 5.60 (95% CI: 1.29–25.00, *p* = 0.009, q = 0.130), indicating that smokers in this cohort were more likely to contract polymicrobial infections than non-smokers. However, neither association was statistically significant after adjustment for multiple testing.

Q3: Monomicrobial streptococcal infections

Twenty-five patients with monomicrobial Streptococcus infection were evaluated. In twelve of these patients with monomicrobial infections, a history of trauma was documented, which likely served as an infection entry point. In univariate analysis, trauma was associated with monomicrobial streptococcal NF, with an OR of 3.40 (95% CI: 1.04–11.70, *p* = 0.029, q = 0.500), but this association was not statistically significant after adjustment for multiple testing.

Q4: Specific Streptococcus groups

This association was also observed when analyzing NF due to *Streptococcus pyogenes* (Group A Streptococcus) (OR 8.10; 95% CI: 2.30–31.90, *p* < 0.001, q = 0.004), indicating a statistically significant association after adjustment for multiple testing.

Q5: Other pathogens

No statistically significant associations between risk factors and other pathogens were identified after adjustment for multiple testing (all q > 0.050).

A body mass index ≥ 30 kg/m^2^, defining obese versus non-obese patients, was not associated with necrotizing fasciitis outcomes.

### Exploratory Multivariable Analysis

Exploratory multivariable analyses using lasso-regularized logistic regression were performed for all research questions with sufficient data.

For hospital death (Q1), the model retained age and female sex, with coefficients of β = 0.23 and β = 0.12, respectively, and an intercept of −1.5. All other variables were shrunk to zero. The model showed limited discrimination, classifying all patients as not experiencing hospital death, with an accuracy of 82% and a maximum predicted probability of 0.28.

For polymicrobial infections (Q2), smoking (β = 0.29), kidney disease (β = 0.14), and liver disease (β = 0.05) were retained, with an intercept of −1.28. The model again defaulted to classifying all patients as negative, with an accuracy of 78% and a maximum predicted probability of 0.36.

For monomicrobial streptococcal infections (Q3), no variables were retained.

For infections with *Streptococcus pyogenes* (Group A Streptococcus) (Q4), trauma (β = 0.42) and heart disease (β = −0.22) were retained, with an intercept of −0.58. This model achieved an accuracy of 78% and identified 11 positive cases with one false positive.

For other pathogens (Q5), including *Escherichia coli*, *Pseudomonas aeruginosa*, *Staphylococcus aureus*, and *Enterobacter cloacae*, no variables were retained.

Overall, the multivariable analyses were consistent with the univariate findings and did not reveal robust predictive patterns in this cohort.

No single λ value is reported due to averaging across cross-validation runs, and AUC was not calculated, as the analyses were exploratory and focused on exploratory variable selection rather than predictive model performance. Reported coefficients are based on standardized predictor variables.

## 4. Discussion

This retrospective, monocentric study aimed to assess the etiology and clinical characteristics of NF within a Swiss university hospital, differentiate risk factors associated with polymicrobial versus monomicrobial infections, and evaluate the effectiveness of diagnostic and therapeutic strategies in improving patient outcomes. Out of 214 patients who initially presented with a referral or clinical suspicion of NF and underwent surgical treatment, only 75 had an intraoperative confirmation of the NF diagnosis, representing 35% of the cases assessed for severe soft tissue infection. This finding highlights the difficulty of diagnosing NF based solely on clinical assessment and suspicion, highlighting the importance of intraoperative confirmation to confirm or exclude NF. It is important to note that severe soft tissue infections can develop a life-threatening course, and surgical intervention for tissue debridement may be indicated regardless of whether they are classified as necrotizing fasciitis. However, the assessment of outcomes after surgical treatment of severe soft tissue infections other than necrotizing fasciitis is beyond the scope of this study.

Our data were collected from a cohort of patients treated over a span of 12 years at a single, geographically centrally located university hospital in Switzerland, providing a valuable basis for extrapolation to the wider Swiss population. The mortality rate observed in our study was 27% (20 deaths out of 75 patients). This rate is relatively low compared to international figures reported in the literature, where mortality rates for NF can reach up to 76% [6]. Possible explanations for this low mortality rate include the consistently radical surgical approach to NF in our clinic as well as the fast emergency referral system in Switzerland with short distances, a highly interconnected healthcare infrastructure, rapid ground and air transport, and a high density of hospitals. All of these factors seem to contribute to rapid diagnosis and timely and appropriate treatment in a specialized center.

However, the hypothesis that mortality in Switzerland would be below 20% could not be confirmed, although the small sample size limits the validity of this finding.

The results yielded several significant associations that align with clinical expectations, while some findings were surprising. The association between advanced age and hospital death is consistent with previous studies [10,35]. In our cohort, 16 of 44 patients over the age of 60 died during hospitalization, which supports the notion that older patients generally have weaker immune systems [36] and heightened vulnerability to severe complications from NF; our data align with existing literature [10,35]. 

However, although several associations were statistically significant in univariate analysis, only the association between trauma and Streptococcus pyogenes remained statistically significant after adjustment for multiple testing. Nevertheless, the observed patterns provide clinically meaningful insights and support the relevance of the identified risk factors, even if statistical significance was not retained after correction.

The exploratory multivariable analysis using lasso-regularized logistic regression provided complementary insights into potential combinations of risk factors. Variables such as age, smoking, kidney disease, and trauma were identified in individual models, supporting their potential clinical relevance. These findings were consistent with the univariate analyses. Given the limited sample size and the rarity of the disease, results should be interpreted with caution and may benefit from confirmation in multicenter studies.

In terms of pathogens associated with mortality, among the 20 patients who died, 15% had polymicrobial infections (3 out of 20 cases) and 75% had monomicrobial infections (15 out of 20 cases). Two additional patients died so shortly after primary surgery that no pathogen could be identified, resulting in 10% of mortality cases with no microbiological data. *Escherichia coli* was the most frequent pathogen among the fatal cases, accounting for 35% of deaths (7 out of 20 cases), with six monomicrobial and one polymicrobial infection.

According to empirical observations, obesity would be expected to be a significant risk factor for both hospital death and infections in patients with NF, as obesity is often associated with further health problems, such as compromised immune function and increased vulnerability to infections [37,38]. However, our data did not support these findings. Despite a notable proportion of obese patients in the sample, no significant association was found between the presence of obesity and the clinical outcomes examined. This discrepancy suggests that the relationship between obesity and NF outcomes may be more complex than anticipated. It is possible that the severe impact of NF overshadows pre-existing differences in health status between obese and non-obese patients, underscoring the need for further investigation in larger cohorts.

Additionally, the relevance of certain comorbidities may vary across populations. For example, diabetes mellitus is more prevalent in the USA [39] than in Switzerland [40], increasing the likelihood of comorbidity with NF in the U.S. population. Consequently, diabetes mellitus may not be as significant a comorbidity for NF in Switzerland [40]. Similarly, the lack of an observed association between obesity and NF in our cohort may reflect Switzerland’s lower obesity rates [41] compared to the US [42]. These findings support the notion that comorbidity patterns in NF can differ by region and population characteristics. Therefore, our finding that obesity is not a significant risk factor for NF in this Swiss cohort may not align with results from countries with higher obesity prevalence [29]. Sociodemographic aspects can be linked to access to the healthcare system and thus may influence diagnostic and treatment outcomes.

Notably, kidney disease was relatively prevalent in the sample, affecting 56% of patients (42 out of 75), yet only 13 of these individuals presented with polymicrobial infections. This suggests that while kidney disease may increase the risk, it is not a deterministic factor, and additional variables may mediate this relationship. Furthermore, the incidence of polymicrobial infections in patients with kidney disease may be attributable to poor wound healing, breaches in skin integrity, and repeated exposure to potentially colonized healthcare facilities. Smoking, though less common in the cohort (13 patients), showed an association in univariate analysis, indicating its potential as a clinically relevant factor. Together, these findings highlight kidney disease and smoking as clinically relevant risk factors for polymicrobial NF infections, warranting closer attention in patient assessment and management strategies.

### Limitations

While the study has limitations, primarily due to the retrospective nature and small sample size, it is important to recognize that NF is a rare disease, which inherently limits the number of cases available for analysis. Additionally, the emergent and life-threatening nature of the disease makes conducting prospective studies difficult, as immediate intervention is required and no time flexibility for thorough prospective patient recruitment is available. Inconsistent data regarding clinical findings may limit the accuracy of certain observations. A further methodological aspect relates to the handling of missing data. Variables with a high proportion of missing values, including potentially relevant inflammatory markers, were not included in the analysis in order to maintain data quality and avoid introducing additional assumptions. While this approach ensures a robust analysis based on reliably documented variables, it is possible that some clinically relevant information was not captured in the final model. In a retrospective emergency setting, missing data may not be entirely random and could be more frequent in patients with severe or rapidly evolving disease. As no multiple imputation or sensitivity analyses were performed, the findings should therefore be interpreted with appropriate caution. A further methodological aspect is the dichotomization of age and BMI based on WHO criteria to enhance clinical interpretability and comparability with existing literature. While this approach simplifies analysis, it may be associated with some loss of information and a reduced sensitivity to detect more subtle associations. For instance, presence of typical map-like redness of the skin was not consistently documented in all patients, through written records or photographic evidence, and pain documentation may have been subjectively assessed in an emergency setting. Despite these limitations, the findings still provide valuable insights into the clinical presentation of NF, and future prospective studies may help to capture such details more systematically, further refining our understanding of important symptoms.

The decentralized structure of the healthcare system in Switzerland further complicates the accumulation of large patient cohorts, as cases can be distributed across multiple institutions. Despite these challenges, the study offers important insights that contribute to the ongoing understanding of NF and its associated risk factors. An interesting consideration for future research would be to nationally assess mortality rate differences according to residential regions within Switzerland.

Another limitation of the study involves the extended timeframe of data collection, during which surgical care was delivered by multiple surgeons. This variability was especially pronounced among residents, where turnover was frequent. However, the presence of a strong hospital policy remained consistent, ensuring continuity in clinical decision-making. Namely, the treatment strategy for NF, focused on rapid and radical surgical intervention, was applied uniformly throughout. Taken together, while these limitations should be acknowledged, they also reflect the real-world complexities of studying rare, acute conditions like NF.

## 5. Conclusions

This study provides valuable insights into the risk factors associated with mortality and infection outcomes in patients with NF within the population in Switzerland. Swiss healthcare infrastructure may contribute to a lower mortality rate in timely treated patients. Advanced age, trauma, kidney disease, and smoking were associated with clinical outcomes; however, after adjustment for multiple testing, only trauma in association with *Streptococcus pyogenes* remained statistically significant. Notably, elderly patients and those with kidney disease or smoking habits demonstrated higher susceptibility to mortality and polymicrobial infections, while trauma was closely associated with Group A streptococcal infections. Identifying subgroups with a higher mortality risk may help clinicians and surgeons in Switzerland adapt to more aggressive therapeutic regimens to better treat these patients.

## Figures and Tables

**Table 1:** Clinical findings.

Operated	Map-Like Redness Present	Pain Out of Proportion
75/75 (100%)	39/75 (52%)	11/75 (15%)

**Table 2:** Clinical course.

Clinical Course	Median	Range [min–max]	Interquartile Range [25th–75th Percentile]
Length of hospital stay (in days)	22	(0–73)	(13–32)
Number of surgeries per patient	5	(1–14)	(3–8)
Number of revision surgeries during hospitalization	1	(0–9)	(0–2)
Time after admission until cessation of infection progress (in days)	6	(0.2–49)	(3–13)
Time from definitive cessation of infection progress to wound closure (in days)	4	(0–30)	(0–8)
Body temperature on admission (in °C)	37	(35–40)	(37–38)
Temperature postoperatively day 1 (in °C)	37	(35–39)	(37–38)
Temperature postoperatively day 2 (in °C)	37	(35–40)	(37–38)
Temperature postoperatively day 3 (in °C)	37	(35–39)	(37–38)

**Table 3:** Antibiotics change.

		Antibiotics Change	%
	64 of 75	patients had 1 change in antibiotic regimen	85%
of whom	23 of 64	underwent escalation of antibiotic therapy	36%
	49 of 74	patients had 2 changes in antibiotic regimen	66%
of whom	13 of 49	underwent escalation of antibiotic therapy	27%
	30 of 74	patients had 3 changes in antibiotic regimen	41%
of whom	14 of 30	underwent escalation of antibiotic therapy	47%
	11 of 74	patients had 4 changes in antibiotic regimen	15%
of whom	04 of 11	underwent escalation of antibiotic therapy	36%

**Table 4:** Postoperative outcomes.

Wound Closure During Hospital Stay	In-Hospital Death	Recurrence After Treatment Considered Ended
51/75 (68%)	20/75 (27%)	4/75 (5%)

**Table 5:** Patients’ characteristics.

Patients’ Characteristics	Median	Range [min–max]	Standard Deviation
Age (years)	63	(13–92)	16
BMI (in kg/m^2^)	28	(14–63)	8

**Table 6:** Comorbidities.

Comorbidities	Number of Patients	%
Presence of at least one known comorbidity	74/75	99%
Coronary heart disease	46/75	61%
History of kidney disease	42/75	56%
Liver disease	32/75	43%
Lung disease	32/75	43%
History of trauma	24/75	32%
Diabetes mellitus	20/75	27%
History of smoking	15/75	20%
Immunosuppression drugs	14/75	19%
History of alcohol consumption	10/75	13%
History of malignancy	9/75	12%
History of drug abuse	7/75	9%
Peripheral arterial occlusive disease	4/75	5%
Immunocompromising disease	2/75	3%

**Table 7:** Pathogens.

Pathogen	n
**Gram-positive bacteria:**	**49**
*Streptococcus pyogenes* (Group A *Streptococcus*)	23
*Streptococcus dysgalactiae subspecies equisimilis* (Group G *Streptococcus*)	5
*Streptococcus dysgalactiae subspecies equisimilis* (Group C *Streptococcus*)	1
Streptococcus anginosus group	4
*Streptococcus agalactiae* (Group B *Streptococcus*)	1
*Streptococcus species*	1
*Staphylococcus aureus*	9
*Staphylococcus simulans*	1
*Enterococcus faecalis*	2
*Enterococcus faecium*	1
*Enterococcus* species	1
**Gram-negative bacteria:**	**40**
*Escherichia coli*	18
*Escherichia coli* (extended-spectrum beta-lactamase producing)	2
*Klebsiella pneumoniae*	4
*Enterobacter cloacae*	8
*Pseudomonas aeruginosa*	7
*Proteus vulgaris*	1
**Anaerobic bacteria:**	**3**
*Actinomyces funkei*	1
*Actinomyces turicensis*	1
*Gardnerella vaginalis*	1
**Fungi:**	**2**
*Candida albicans*	2

**Table 8:** Antibiotic therapy.

Initial Antibiotic Therapy at Clinical Suspicion of NF	n
Clindamycin	48
Piperacillin-Tazobactam	40
Amoxicillin-Clavulanate	17
Meropenem	11
Vancomycin	10
Ceftriaxone	10
Cefepime	6
Metronidazole	5
Gentamicin	2
Cefazolin	2
Imipenem	1
Benzylpenicillin	1
Ciprofloxacin	1

**Table 9:** Antibiotic therapy combinations.

Most Common Initial Antibiotic Combinations	n
Clindamycin + Piperacillin-Tazobactam	15
Amoxicillin-Clavulanate + Clindamycin	10
Piperacillin-Tazobactam	8
Clindamycin	5
Clindamycin + Piperacillin-Tazobactam + Vancomycin	3
Other combinations	33

## Data Availability

The datasets used and/or analyzed during the current study are available from the corresponding author on reasonable request.

## Abbreviations

BMI	Body-Mass Index
CRP	C-reactive protein
FDR	False discovery rate
LRINEC	Laboratory Risk Indicator for Necrotizing Fasciitis
NF	Necrotizing Fasciitis
OR	Odds Ratio
USA	United States of America
